# Mediating Role of Trait Mindfulness on the Relationships Between Age and Both Depressive and Anxiety Symptomology

**DOI:** 10.1093/geroni/igaa057.1195

**Published:** 2020-12-16

**Authors:** Scotti Howard, Eric Allard

**Affiliations:** Cleveland State University, Cleveland, Ohio, United States

## Abstract

Previous research has shown that despite experiencing more negative life events, older adults maintain relatively high levels of well-being compared to their younger counterparts. This effect appears to be at least partially mediated by trait mindfulness in older adults (Raes et al., 2013). The current study expanded into an investigation as to how trait mindfulness might intervene on the relationship between age and other well-being indicators: anxiety and depressive symptomology. Participants included 30 older adults (aged 60-83) and 41 young adults (aged 18-35). Trait mindfulness was examined using the Mindful Attention Awareness Scale (MAAS), while depressive symptoms and trait anxiety were measured using the Center for Epidemiological Studies Depression Scale (CES-D) and the State-Trait Anxiety Inventory (STAI), respectively. Two separate mediated multiple regression models were conducted using Hayes’ PROCESS Macro in SPSS. Trait mindfulness exhibited a significant indirect effect on the relationship between age and depressive symptoms (β = -2.27, p < .005), which was also seen for the relationship between age and trait anxiety (β = -4.17, p < .001). Older age predicted higher trait mindfulness, which in turn predicted diminished self-reported anxiety and depressive symptomology. Controlling for mindfulness in these models reduced the direct effect of age on depression and anxiety to non-significance. These findings imply that the relationship between age and trait mindfulness can be extended to alternative markers of well-being.

